# Oral cancer: Clinicopathological features and associated risk factors in a high risk population presenting to a major tertiary care center in Pakistan

**DOI:** 10.1371/journal.pone.0236359

**Published:** 2020-08-06

**Authors:** Namrah Anwar, Shahid Pervez, Qurratulain Chundriger, Sohail Awan, Tariq Moatter, Tazeen Saeed Ali

**Affiliations:** 1 Department of Pathology and Laboratory Medicine, Aga Khan University Hospital, Karachi, Pakistan; 2 Department of Otolaryngology, Head and Neck Surgery, Aga Khan University Hospital, Karachi, Pakistan; 3 School of Nursing and Midwifery, Aga Khan University Hospital, Karachi, Pakistan; University of Cincinnati College of Medicine, UNITED STATES

## Abstract

Oral squamous cell carcinoma (OSCC) has the highest prevalence in head and neck cancers and is the first and second most common cancer in males and females of Pakistan respectively. Major risk factors include peculiar chewing habits like areca nut, betel quid, and tobacco. The majority of OSCC presents at an advanced stage with poor prognosis. On the face of such a high burden of this preventable cancer, there is a relative lack of recent robust data and its association with known risk factors from Pakistan. The aim of this study was to identify the socioeconomic factors and clinicopathological features that may contribute to the development of OSCC. A total of 186 patients diagnosed and treated at a tertiary care hospital, Karachi Pakistan were recruited. Clinicopathological and socioeconomic information was obtained on a structured questionnaire. Descriptive analysis was done for demographics and socioeconomic status (SES) while regression analysis was performed to evaluate the association between SES and chewing habits, tumor site, and tumor stage. The majority of patients were males and the mean age of OSCC patients was 47.62±12.18 years. Most of the patients belonged to low SES (68.3%) and 77.4% were habitual of chewing. Gender (male) and SES were significantly associated with chewing habits (p<0.05). Odds of developing buccal mucosa tumors in chewers (of any type of substance) and gutka users were 2 and 4 times higher than non-chewers respectively. Middle age, chewing habits, and occupation were significantly associated with late stage presentation of OSCC (p<0.05). In conclusion, male patients belonging to low SES in their forties who had chewing habits for years constituted the bulk of OSCC. Buccal mucosa was the most common site in chewers and the majority presented with late stage tumors.

## Introduction

Cancer is by far the first and second most common cause of death in developed and developing countries respectively [1]. The GLOBOCAN 2018 presented an estimation of 18.1 million new cases of cancer and 9.6 million deaths from cancer in 2018 [2], which was 14.1 and 8.2 million respectively in 2012. Amongst all the cancers, Head and Neck Squamous cell carcinoma (HNSCC) presents with 600,000 cases worldwide, with 40–50% mortality annually and the burden is estimated to almost double in developing countries by 2030 [3]. Most of these tumors arise from the epithelial cells of oral cavity (OC), oropharynx, larynx or hypopharynx [4]. Oral squamous cell carcinoma (OSCC) has the highest prevalence in the HNSCC group that has shown to be 11^th^ and 18^th^ most common cancer worldwide as per 2012 and 2018 data respectively. This overall global decrease in the prevalence of OSCC is attributed to the lesser chewing habits and geographic heterogeneity, however, it is still the most common cancer in South Asia, South Central Asia as well as the Pacific Islands (Papua New Guinea, with the highest incidence rate worldwide in both sexes). In 2018, India alone had estimated 120,000 new patients diagnosed, of which about 72,000 patients died [5]. Taiwanese region has also presented with the world's highest incidence rates of OC cancer which accounted for 8% of all new cancers diagnosed and 6.3% of all cancer deaths in 2014 [6]. In the subcontinent, this cancer ranks first in Bangladeshi and Pakistani males (9).

The anatomy of OC is critical because of interrelated structures and for long time carcinomas of oral cavity and oropharynx were grouped as OSCCs which also changed the epidemiological data [7]. Advanced translational and clinical researches have now been able to differentiate both in many aspects [8]. Tumors of oropharynx involve the base of tongue, palantine tonsils, soft palate and adenoids [9] whereas, OC starts from the vermillion of the lips and extends posteriorly to circumvallate papillae of the tongue including the alveolar ridge and gums, the anterior two-thirds of the tongue, floor of the mouth, buccal mucosa, retromolar trigone, and hard palate [10]. Amongst OSCC sites, tongue and buccal cavity cancers are more common followed by lip and palate [7]. The risk factors of OSCC vary with geographic location which include smoking, alcohol, variable chewing habits, and infection with high-risk human papillomaviruses (HPVs) among others. Lip and OC cancer were the 2^nd^ most common cancer in Pakistan if both genders are combined (10.9%) and first in males with 15.9% new cases. This increase in the burden is associated with the increased use of areca nut or any type of smokeless tobacco (SLT). SLT is a group of more than 30 products that differ in their toxicity and addictiveness depending upon the composition [11]. The classic chewing substances are betel quid (synonym with paan contains betel leaf, slaked lime, areca nut, and tobacco), gutka (areca nut, tobacco, paraffin wax, slaked lime, and any flavoring) pan masala (slaked lime, areca nut, tobacco, musk ketones), naswar (tobacco, ash, lime), tobacco, mainpuri, and mawa (tobacco, lime, and areca nut) [12]. These substances contain around 28 known carcinogens amongst which arecoline, nonvolatile alkaloid-derived, nitrosamines, volatile aldehydes, flavonoids and tannins are of prime importance [13, 14]. All these chemical compounds change the normal morphology of the cells thus inducing cytogenetic or genetic alternations. Low socioeconomic status (SES) has predictive importance specifically for HNSCC and an increased incidence has been reported in low-income countries [15–17]. In the local context, the frequency of chewing habits has also been shown to be associated with low socioeconomic backgrounds, education, and is more prevalent in males [18, 19]. Challenged with such a high burden of OSCC in our population, there is a relative lack of information regarding the prevalence and association of these habits with OSCC and low SES in recent times. This study aimed to estimate the association of socioeconomic factors, chewing habits including frequency of chewing, smoking as well as location and family history with clinicopathological features of OSCC in a subset of high risk Pakistan population.

## Methodology

### Study design

Cross-sectional study design was used where the population was defined as those who had developed OSCC. A total of 186 OSCC diagnosed patients of 2017 were recruited. Sample size of the study was calculated by Epi info 7 while considering population as unknown; the following assumptions were used for sample size calculation; 80% power, 0.05 significance level with odds ratio (OR) of 2, and 30% expected proportion of OC patients without chewing habits.

### Inclusion/exclusion criteria

Inclusion criteria: Only those patients were recruited who were diagnosed and treated for OSCC at Aga Khan University Hospital (AKUH), a tertiary care hospital in Karachi Pakistan, and gave their informed consent at the time of surgery for their sample to be used for the research purposes. People of any age were included in the study. Consent was also taken before asking the questions and in case of deceased, consent and information were obtained from an immediate family member.

Exclusion criteria: Anyone not meeting the inclusion criteria or people who refused to provide information and with missing contact information were excluded.

### Ethical approval

Under an Institutional Review Board approval at AKUH, ethical approval for the current study was obtained (via its letter 4091-Pat-ERC, dated June 15, 2016).

### Data collection

The list of OSCC patients after their surgery at Aga Khan University Hospital was obtained from Department of Health Information Management Services (HIMS). Initial information of 195 patients about medical record number, patient contact information, tumor site, and date of the surgery was gathered from the list obtained and screening of patients was done as per inclusion criteria. Detailed pathology reports were reviewed to acquire information regarding tumor stage, grade, and site. Socio-demographic details including, age, marital status, location, educational level, occupation, social status, chewing habits, history of cancer in the family, frequency of chewing and, type of chewing substance were directly collected from patients on a structured questionnaire. Out of 195, eight patients could not fulfill the criteria and were excluded leaving the total sample size of 186. Amongst the variables, ‘Occupation status’ was used to define if there are any means of earning a living and as a key measure of socioeconomic status whereas, ‘Occupation’ further elaborated type of work. Participants were asked about the type of occupation and it was categorized into “labor” (any kind of unskilled or skilled manual labor), any kind of business (vendor to factory owner), and office/desk work. Income stratification was based on World Bank classification where income below $1.90 per day is considered as low-class income and the range was set according to current contextual socioeconomic conditions [20, 21]. Participants who had the habit of chewing any substance for at least six months to one year at any point in their life were considered as ‘chewers’ and frequency was defined as number of quid or substances chewed per day [22, 23]. All types of chewing substances used by patients were included and frequency was taken as 1–5, 6–20, and more than 20 times per day. Cigarette smoking was considered habitual if an individual had been smoking for more than a year and was further divided based on per day consumption into casual/light smoker, i.e., < 5 cigarettes/day while heavy smokers were further divided in two categories i.e., < or equal to one pack (20 cigarettes)/day and > one pack or 20 cigarettes/day [24]. Tumor sites were grouped into buccal and non-buccal (including lip, tongue, palate, retromolar trigone). The staging was done according to AJCC 8^th^ Edition criteria and grouped as Early stage (I, II) and Late stage (III, IV). For oral cancer, tumors localized to the organ or site of origin are classified in stages I and II. Local extension of the primary tumor or spread to regional lymph nodes changes the stage to III and IV. In the case of OSCC, classification of category T has been revised according to the “depth of invasion (DOI)”. If the tumor thickness is ≤2 cm with DOI < 5 mm that is T1, but if the same tumor has DOI >5 mm and ≤10 mm, it will be T2. Similarly, T3 would be a tumor of size > 4 cm and DOI ≤ 10 mm, but if DOI becomes >10 mm, T classification upstages to T4 regardless of the tumor size. Nodal status (N) is categorized into N0 = no regional lymph node metastasis, N1 = metastasis in single ipsilateral node ≤3 cm without ENE, N2 = metastasis in a single ipsilateral node which is either ≤3cm with ENE or >3 cm but <6 cm without ENE, or metastasis in multiple ipsilateral or contralateral/bilateral lymph nodes without ENE, none of which is >6 cm. N3 = metastasis in a lymph node >6 cm without ENE or single ipsilateral node >3 cm with ENE or multiple ipsilateral, contralateral or bilateral lymph nodes of any size with ENE. Metastasis (M) is classified into M0 (no distant metastasis) and M1 (distant metastasis) [25].

### Data analysis

Data were analyzed using the SPSS package 20 (IBM, Rochester, USA) for the association and variables included were age, gender, patient current condition, marital status, location, educational level, type of occupation, monthly income, chewing habits, frequency of chewing, type of chewing substance, smoking, frequency of smoking, history of cancer in the family, tumor stage, and tumor site. Descriptive properties were checked by the frequency table of all psychosocial factors and in case of using multiple chewing products by one patient, each product was considered separately. Odds ratios and their respective 95% confidence intervals (CI) were estimated using univariate and multivariate logistic regression taking tumor site, chewing habit, and tumor stage as dependent variables. All analyses were set as two-sided and a p-value less than 0.05 was considered significant. For analysis purpose values in some variables were grouped based on the overall data trend. For logistic regression people with unknown job status, retired, unemployed and housewives were grouped into “unemployed” and laborers, businessmen (factory, shop, and stall), and daily wagers were grouped as “employed”. Primary, middle, and matric education was grouped as “Primary to Matriculation”. Intermediate (equivalent to 12 years of high school) and diploma were grouped and with unknown education level were assumed uneducated.

## Results

Socio-economic status (SES) was determined by descriptive analysis and by performing univariate and multivariate logistic regression, odds ratios were calculated to determine the association between dependent variables and other variables. Table 1 illustrates the baseline socio-demographic characteristics of the patients. Overall, 186 patients sample were taken, out of which 149 were males, and 37 were female (4:1 ratio). The participants’ mean age was 47.6 years, amongst which 80.1% were alive (both genders). The population of the study comprised of different occupations in which 30.1% were laborers, 22.6% were businessmen, and 18.3% were working in the office. Amongst this population, most of the people belonged to the group of earning <$120 (PKR20,000), 36% of the participants' monthly income was between $120–250 (PKR20,000 and 40,000) while only 18.8% participants’ had monthly income >$ $380 (PKR 60,000). The population was also analyzed based on their location and address, in which 59.1% were from Karachi (the capital city of province Sindh), 20.4% from Hyderabad (a twin city of 165km from Karachi) and 10.7 and 9.7% were residents of interior Sindh and other than Sindh, respectively. Based on the education level, 13.4% and 23.1% had intermediate (higher secondary school) and graduation level of education, respectively, while 28% were uneducated. Amongst the participants, only 22% were found to be smokers, in which 19.5% were chain smokers who smoked more than 20 cigarettes per day (1 pack). It was found that 77.4% of the participants had habit of chewing in any form. Some of the patients were observed to have chewing habits for more than one type of substance. This resulted in a larger summation of this variable than the sample size, which was adjusted by logistic regression analysis. In this cohort, less than half of the participants (43%) were habitual of betel quid (paan) followed by gutka. Frequency of chewing any type of SLT or areca nut clustered mostly around 5 times per day while 24.3% chewed almost 20 times per day. The participants were assessed for the cancer history in the family, 16.7% of the participants had a family history of cancer, and 12.9% with OC cancer while rest had lung or other types of cancers. Based on the treatment types in which 39.2% of the participants underwent multimodality i.e., surgery, chemotherapy, and radiotherapy. For the site of tumor, 68.8% had buccal mucosal cancer while 67.2% had Grade II (moderately differentiated) tumors. Moreover, 79% of the participants presented with late stage of cancer.

**Table 1:** Descriptive analysis for demographics and Socioeconomic status (SES) and clinicopathological features of OSCC. N = 186.

	Characteristics	Frequency (n)	Percentage (%)
**1.**	**Gender**		
	Male	149	80.1
	Female	37	19.9
**2.**	**Mean age of the Participants = 47.62±** **12.18**		
**3.**	**Patient current status**		
	Alive	149	80.1
	Dead	37	19.9
**4.**	**Occupation status**		
	Employed	132	71
	Unemployed	54	29
**5.**	**Type of occupation**		
	Labor	56	30.1
	Businessman	42	22.6
	Office worker	34	18.3
	Housewife	35	18.8
	Retired	9	4.8
	Not defined	10	10.2
**6**	**Monthly income of family**		
	Less than $120(<PKR 20,000)	84	45.2
	$120–250 (PKR 20,000–40,000)	43	23.1
	$250–380 (PKR 40,000–60,000)	24	12.9
	More than $380 (>PKR 60,000)	35	18.8
**7**	**Location**		
	Karachi	110	59.1
	Hyderabad	38	20.4
	Interior Sindh	20	10.8
	Other than Sindh province	18	9.7
**8**	**Marital Status**		
	Married	171	91.9
	Unmarried	15	8.1
**9**	**Education**		
	Uneducated	52	28.0
	Primary till Matriculation	66	35.5
	Intermediate/Diploma	25	13.4
	Graduation/Master	43	23.1
**10.**	**Smoking Status**		
	Yes	41	22.0
	No	145	78
**11.**	**How many cigarettes do you smoke per day?**		
	Casual (< 5 cigarettes per day)	13	31.7
	< than 1 pack per day (20 cigarettes)	20	48.8
	>than 1 pack per day	8	19.5
**12.**	**Addiction to Chewable tobacco**		
	Yes	144	77.4
	No	42	22.6
**13.**	**Mean age of patients with habit of chewing =** 46.2± 11.8		
**14.**	**Mean age of patients without habit of chewing =** 52.4± 12.3		
**15.**	**Type of Chewable tobacco**		
	Betel quid (paan)	80	43.0
	Gutka	59	31.7
	Mawa	4	2.2
	Naswar	9	4.8
	Areca nut (chalia)	44	23.7
	Tobacco	31	16.7
**16.**	**Frequency of Chewable Tobacco**		
	Less than 5 times a day	72	50.0
	6–19 times a day	37	25.7
	≥ than 20 times a day	35	24.3
**17.**	**Family History of Cancer**		
	Yes	31	16.7
	No	155	83.3
**18.**	**Diagnosis of the family members having cancer**		
	Oral Cancer	4	12.9
	Lung Cancer	1	3.2
	Other Cancer types	26	83.9
**19.**	**Treatment of Participant**		
	Surgery	41	22.1
	Chemotherapy	2	1.1
	Radiotherapy	9	4.8
	Surgery + Chemotherapy	9	4.8
	Surgery + Radiotherapy	25	13.5
	Chemotherapy + Radiotherapy	27	14.5
	Surgery + Chemotherapy + Radiotherapy	73	39.2
**20.**	**Participant Site of Tumor**		
	Buccal Mucosa	128	68.8
	Non-buccal Mucosa	59	31.7
**21.**	**Grade of the Tumor**		
	Grade I	39	20.6
	Grade II	127	67.2
	Grade III	23	12.2
**22.**	**Stage of Tumor**		
	Early Stage (I,II)	39	21.0
	Late Stage (III, IV)	147	79.0

Univariate logistic regression analysis of SES with chewing habits indicated the association (Table 2). At CI 95%, OR>1, and p< 0.05 the variables were considered to be significant. Males were 2.2 times more likely to be chewers than females (CI: 1.02–4.93). In income, the highest odds of chewing were observed in the group of $120–250 [OR: 3.644, (CI: 1.21–10.97)], whereas people with income above $250 were less likely to have chewing habits. People with basic level education (primary to high school) were 4.2 times more likely to be chewing followed by higher secondary school education [OR: 3.433, (CI: 1.00–11.76)]. Marital status, smoking habits (and frequency), family history of cancer, type of cancer in the family, and kind of occupation did not show any association with the chewing habits. Multivariate analysis was performed for possible confounding factors (Table 2, Model 1). The model was adjusted for age, gender, patient status, marital status, and monthly income due to the biological importance of the variables. An insignificant p-value of chi-square in Hosmer and Lemeshow Test validated the goodness-of-fit of the model. For chewing habits of OSCC patients, the factors statistically significant included education record; either primary to matric or uneducated (adjusted OR 5.876, CI: 1.61–21.45, adjusted OR 6.323, CI: 1.55–25.70) and location.

**Table 2:** Model 1, univariate and multivariate regression analysis of SES variables with chewing habits as dependent variables in OSCC patients.

	Factors	Chewing habits (Yes) n (%)	Chewing habits (No) n (%)	OR (95% CI)-univariate	Adjusted OR (95% CI)-multivariate
**1.**	**Age**	46.2±11.8	52.4±12.3	1.0 (1.01–1.05)	
**2.**	**Gender**				
	Female	24 (64.9)	13(35.1)	1(Reference)	
	Male	120 (80.5)	29(19.5)	2.2 (1.02–4.93)	
**3.**	**Monthly income**				
	>$380 (PKR 60,000)	22(62.9)	13(37.1)	1	
	$120–250 (PKR 20,000–40,000)	37(86.0)	6(14.0)	3.6 (1.21–10.97)	
	< $120 (PKR 20,000)	68(81.0)	16(19.0)	2.5 (1.05–6.03)	
**4.**	**Location**				
	Other than Sindh	9(50.0%)	9(50.0%)	1	
	Karachi	89(80.9)	21(19.1)	4.2 (1.50–11.98)	
	Hyderabad	34(89.5)	4(10.5)	8.5 (2.12–34.06)	17.0 (2.54–114.52)
**5.**	**Education**				
	Graduation & above	26(39.5)	17(60.5)	1	
	Inter/Diploma	21(84.0)	4(16.0)	3.4 (1.00–11.76)	
	Primary to Matric	57(86.4)	9(13.6)	4.1 (1.63–10.51)	5.8 (1.61–21.45)
	None	40(76.9)	12(23.1)	2.1(1.00–5.30)	6.3(1.55–25.70)

-Only significantly associated variables are presented in the table, OR 1 is taken as Reference, p<0.05 as significant.

-Adjusted for age, gender, patient status, marital status, and monthly income due to the biological importance of the variables in multivariate analysis.

The association of psychosocial factors and SES was analyzed with the site of tumor (buccal and non-buccal) by univariate and multivariate logistic regression. Table 3-Model 2 presents the significantly associated variables (p<0.05, OR >1) with the tumor site of OSCC patients. Males were again 3 times more likely to have tumor of the buccal cavity than females (CI: 1.42–6.25). People with a monthly income of $120–250 showed OR of 3.6 (CI: 1.33–10.18) whereas, other income categories were not significantly associated with tumor site. People with primary to matric education had 3.2 times more odds of having buccal tumor (CI: 1.37–7.59). Amongst the types of chewable substances, only gutka was found to be significantly associated with the buccal cavity [OR: 4.140, (CI: 1.81–9.45)]. People of blue-collar occupation (labor, businessman, and daily wages) had the highest odds of having tumor of buccal site than others. [OR: 2.586, (CI: 1.33–5.02)]. In multivariate Table 3-Model 2, age, patient status, marital status, occupation, and education were kept in the adjusted variables and insignificant p-value for goodness-of-fit validated the model. In multivariate analysis, factors statistically significant included being male: (adjusted OR 4.150, CI: 1.57–12.90), monthly income [either $120–250 (adjusted OR 4.987, CI: 1.68–14.81) or <$120 (adjusted OR 4.434, CI: 1.49–13.16)], and gutka chewing: (adjusted OR 3.063, CI: 1.27–7.41). Age, patient current condition, occupation status, marital status, smoking, chewing frequency, types of chewing substance (apart from gutka), and family history of cancer were shown not to be associated with the tumor of buccal cavity site. Location gave the broad CI with both chewing habits [location Karachi (CI: 2.32–58.8)/Hyderabad (2.54–114.52)] and tumor site [Location Karachi: (adjusted OR 10.223, CI: 1.65–63.46)], though p-values were significant.

**Table 3:** Model 2, univariate and multivariate regression analysis of SES variables with tumor site as dependent variable in OSCC patients.

	Factors	Buccal Cavity n (%)	Non-Buccal Cavity n (%)	OR (95% CI)-univariate	Adjusted OR (95% CI) -multivariate
**1.**	**Gender**				
	Female	18(48.6)	19(51.4)	1 (Reference)	
	Male	110(73.8)	39(26.2)	2.9 (1.42–6.25)	(1.57–12.90)
**2.**	**Monthly income**				
	> $380 (PKR 60,000)	19(54.3)	16(45.7)	1	
	$120–250 (PKR 20,000–40,000)	35(81.4)	8(18.6)	3.6 (1.33–10.18)	4.9 (1.68–14.81)
	< $120 (PKR 20,000)	58(69)	26(31)	1.8 (0.84–4.22)	4.4 (1.49–13.16)
**3.**	*Location				
	Other than Sindh	10(55.6)	8(44.4)	1	
	Karachi	73(66.4)	37(33.6)	7.2 (1.27–40.68)	10.3 (1.65–63.46)
**4.**	*Education				
	Graduation & above	24(55.8)	19(44.2)	1	
	Primary to Matric	53(80.3)	13(19.7)	3.2 (1.37–7.59)	
**5.**	*Gutka chewing				
	No	77(60.6)	50(39.4)	1	
	Yes	51(86.4)	8(13.6)	4.1 (1.81–9.45)	3.0 (1.27–7.41)
**6.**	**Occupation**				
	Unemployed	29(53.7)	25(46.3)	1	
	Employed	99(75)	33(25)	2.5 (1.33–5.02)	

-OR 1 is taken as Reference, variables with p<0.05 were considered significant and are presented in the table.

-*Other categories of location, education and type of chewing product were not significantly associated hence are not presented.

- Patient status, marital status, occupation, and education were kept in the adjusted model due to the biological importance of the variables in multivariate analysis.

When checked for the association between tumor stage (early stage I/II and late stage III and IV), SES and psychosocial factors; age, chewing habits, type of chewing substance (areca nut) and occupation were significantly associated with the late stage tumors (Table 4-Model 3) middle-aged people (45.9 years of age) were likely to have more odds of presenting with late stage of cancer (CI: 1.02–1.09). People with chewing habits (of any substance) had 2.3 times more odds of developing late stage cancer (CI: 1.10–5.16), p-0.028]. Among the types of chewing products, only areca nut consumers came out to be 4.6 times more likely to have late stage cancer (CI: 1.35–15.91). Occupation (employed) showed OR of 2.11 (CI: 1.13–4.82). This model also provided goodness-of-fit and multivariate analysis was performed for tumor stage of OSCC patients and the factors statistically significant were age (adjusted OR 1.049, CI: 1.01–1.07), areca nut chewers; (adjusted OR 5.417, CI: 1.45–20.18) and model was adjusted for gender, patient status, marital status, location, education status, and monthly income.

**Table 4:** Model 3, univariate and multivariate regression analysis for significant association of tumor stage of OSCC patients with SES.

	Factors	Early stage tumor n (%)	Late stage tumor n (%)	OR (95% CI)-univariate	Adjusted OR (95% CI) -multivariate
**1.**	**Age**	53.7±14.2	45.9±11.1	1.06 (1.02–1.09)	1.04 (1.01–1.08)
**2.**	**Chewing habits**				
	No	14(33.3)	28(66.7)	1 (Reference)	
	Yes	25(17.4)	119(82.6)	2.3 (1.10–5.16)	
**3.**	*Areca nut chewing				
	No	36(25.4)	106(74.6)	1	
	Yes	3(6.8)	41(93.2)	4.6 (1.35–15.91)	5.4 (1.45–20.18)
**4.**	**Occupation**				
	Unemployed	16(29.6)	38(70.4)	1	
	Employed	23(17.4)	109(82.6)	2.1 (1.13–4.82)	

-OR 1 is taken as Reference, variables with p<0.05 were considered significant and are presented in table.

-* Other categories of types of chewing products did not show any significance.

-Multivariate model was adjusted for gender, patient status, marital status, location, education status, and monthly income.

## Discussion

Pakistan particularly Southern Pakistan with its capital Karachi, largest city of Pakistan, and 6^th^ most populous (over 20million population) in the world has shown a stunning increase in OSCC over the years that is because of excessive use of different chewing products amongst various age groups. In this study, age showed significant association with chewing habits that concurs with some studies published from Pakistan and India, reporting the mean age of the patients to be between 41–50 years [26–28]. In India, according to the Global Adult Tobacco Survey (GATS, 2009–2010), one-third of the Indian population ≥15 years of age use tobacco in some form [29]. People between 15 and 49 years of age are habitual of using some form of tobacco and 32.9% and 18.4% of men and women respectively, are smokeless tobacco users [5]. According to a collaborative study in South and East Asia, chewing was more prevalent among men (10.7–43.6%) in Taiwan, Mainland China, Nepal, and Sri Lanka whereas, in women, rates were high in Malaysia and Indonesia (29.5–46.8%) [29]. This partially holds with this study where the male to female ratio was 5:1 for chewing habits.

Education plays an important role in the overall health of a country in terms of awareness toward the use of hazardous substances. Considerable regional variation was reported in both the prevalence and the number of chewing substance users with a higher percentage in LICs and LMICs. The prevalence of chewing was as high as 22% in Southeast Asia; it was <1% in the Western Pacific region [30]. In the South-Asian region, OSCC is highly associated with low socioeconomic status, where people are less aware of the consequences of carcinogenic compounds because of less education. The main reason is chewing areca nut related products particularly in low SES, where it is believed to increase work capacity, alertness, suppress hunger, and are a cheap source of entertainment [31]. According to a recent systematic review, tobacco use in low income, caste, and socioeconomic status groups was roughly twice that of high-status groups [16]. Our research coincides with the studies showing that most of the people having OSCC belonged to low SES and were habitual of chewing (mostly blue-collar workers, servicemen, farmers or housewives). A somewhat similar study in India found that most of the OSCC recruited patients were middle-aged, belonged to low SES, and were habitual of chewing [27]. In a combined study from India and Bangladesh, chewing was associated with less education and low income [32]. In Bangladesh, over half of the habitual chewers have no proper education (secondary level education) with more prevalence in rural areas [33, 34]. Studies from Myanmar have shown more than 50% prevalence of chewing and attributed to poor knowledge and low education [35, 36]. As reviewed by Keith et al., the use of any form of chewing substances varies regionally (from Asia to Africa) and is mostly associated with low SES [37]. This applies to our study, where less education was directly proportional to chewing and implies that gender is an independent factor. However, multiple regression models did not show any association between sex, marital status, and education with the frequency of betel quid use [31] which holds for our study.

The OC subsites represent a variety of trends in the onset of cancer, type of cancer, and association with the consumption of different carcinogenic substances or genetic predispositions. OSCCs are the aggressive malignant lesions that commonly metastasize to local regional lymph nodes and adjoining tissues [38]. In our population odds of OSCC in buccal mucosa cavity were two times higher in the chewers and it was strongly associated with males and low SES which is supported by different regional studies reporting buccal cavity as the most common site [27, 39, 40]. As reported by Siddiqi and colleagues, the relative risk to develop mouth (oral cavity, tongue, and lip) cancers was 3.43 in chewers/SLT users [11]. In western countries, tongue is the most common site of OSCC because of excessive smoking and drinking alcohol [41]. One possible reason is the direct contact of the cigarette with tongue during smoking while rest of the components are inhaled, whereas, the chewing products are kept in the mouth for a longer period. In the descriptive analysis of this study betel quid usage was highest followed by gutka, however, in univariate and multivariate analysis gutka users appeared to have the highest odds of developing buccal mucosa cancer. Rationally, gutka is placed between the teeth, held against the buccal mucosa for a longer duration, and lightly chewed and sucked occasionally. Gutka users consume more dry weight of tobacco, areca nut, and slaked lime as compared to betel quid users and betel leaf also has some anticarcinogenic properties lacking in gutka [42]. The chemical composition of all these chewing substances is shown to have high cytotoxic and genotoxic effects *in vitro* and *in vivo* [43]. Keeping these substances in the mouth for a long time causes mechanical friction in buccal mucosa leading to premalignant lesions or invasive cancer. The frequency of any substance chewed per day did not show any significant association with the tumor site and majority users took these 1–5 times/day and for almost 20 years which concurs with another study from Bangladesh [31].

A large proportion of overall OSCC patients are usually not diagnosed until their disease has reached an advanced stage, ultimately requiring aggressive treatment and relapsing in 50% cases [44]. In this study tumor stages were grouped into two i.e., Early stage (stage I, II) and Late stage (III, IV). Results from this study imply the association of low SES with chewing habits and late stage presentation supported by different studies. Areca nut is the fourth most common psychoactive substance used in the world [45] and habitual areca nut chewers present with more aggressive cancer phenotypes, high rates of metastasized cancer, recurrence, and poor patient survival [46]. Though the association of areca nut chewing with late stage tumor remains largely unexplored, this is just an observation with no definite answers. However, it is speculated that people tend to keep areca nut in the mouth for a longer period simply because it is hard wood-like stuff which does take a long time to chew, and in contrast to other products, its extract is sucked. As little as microgram quantity of this extract has shown to be needed for any cytotoxic or genotoxic event resulting in disease progression [45, 47]. Patients with low SES are not only at higher risk of developing OSCC, but are also to be diagnosed at late stage [48]. In a recent study from India, 82.15% OSCC cases were significantly presented in the later stage [49]. A study by Chu et al., in Asian and Pacific Islanders showed that patients with low SES were most likely to be diagnosed with more advanced disease the first time [50]. In an American study recruiting Afro-Americans stated an association between SES, job status, and marital status on one side and delayed diagnosis on the other [51]. A multi-ethnic study including Malays, Indians, and Indigenous people showed the presence of late stage tumors to be 72.5–78.0%, whereas the Chinese displayed widespread distribution trend of all the stages. The 68.2% Indian and 70.7% Malay patients presented with T3 and T4 tumors [52]. Another study from Taiwan showed 49.3% of patients' habit of chewing and 50.3% of chewing/smoking presented with late stage cancer [53]. In Pakistan, unfortunately, however, most cancer patients tend to present at late stages for a variety of reasons like poverty, lack of awareness, inaccessibility to affordable healthcare, fear of social fall out, etc.

Smoking in our study did not show any association with either chewing habits, tumor site or tumor stage, and only 22% of the patients were habitual smokers. Despite its well know association with HNSCC, it did not show independent predictive significance as 5 years predictor of secondary primary tumors of OCSCC [54]. Smoking has been presented to have a synergistic effect with chewing and alcohol consumption in different studies, but subsites most commonly affected are oropharynx or larynx than oral cavity [55–58]. Anatomically turbulent respiratory flow exposes larynx and pharynx more to the cigarette smoke as compared to OC [57]. Some patients were habitual of using multiple chewing products, having a mixture of several ingredients over and above widely variable chewing duration and quantity of every product in our cohort, which could have a synergistic effect. Other factors, i.e., family history, type of cancer in the family, and marital status did not show any significance with the dependent variables elucidating that chewing habits, tumor site, and tumor stage are less likely to be affected by any of these variables.

## Limitations

This study elaborates on the chewing habits with SES and clinicopathological features of OSCC in recent times in local context; nonetheless, this study had certain limitations, and first is the sample size and duration of the study. Confidence interval in the analysis was broad, implying the associations were not causal rather had confounders, which were though adjusted in the multivariate analysis. Still, a large sample size would provide more narrow ranges. Studies have been done to check the dose-dependent effect of chewing and smoking with OSCC [22], more precise details about pack numbers, chew years, and quit years (if applicable) could be obtained to strengthen the quantification and dose-dependent effects of these substances. We did not determine the survival rate after the first diagnosis and studies report that early stage OSCC has better five-year survival than late stage [59–61].

## Conclusion

Areca nut related products have been declared as type I carcinogen by IARC [12] and this study showed the link between chewing, low SES, and OSCC. Chewing habits are the foremost cause of a drastic increase in OSCC from this region consequently changing the normal mucosa of the oral cavity leading to cancer. This is a comprehensive study conducted on a number of patients, visiting one of the largest tertiary care Hospitals in Pakistan, minimizing selection bias. Despite the recent increase in the price of tobacco products, the consumption is overall increasing even in the low socio-economic group with meager income. This study could provide an insight into ongoing evidence and the causes of oral cancers, which would help in taking measures to control this chewing endemic in Pakistan by educating people, spreading awareness about the hazardous effects and severity of the disease.

## Abbreviations

OC: Oral cavity
OSCC: Oral squamous cell carcinoma
HNSCC: Head & neck squamous cell carcinoma
SES: Socioeconomic status
SLT: Smokeless tobacco
LIC: Low income country
LMIC: Low middle income country
